# Deciphering the streamlined genome of *Streptomyces xiamenensis* 318 as the producer of the anti-fibrotic drug candidate xiamenmycin

**DOI:** 10.1038/srep18977

**Published:** 2016-01-08

**Authors:** Min-Juan XU, Jia-Hua WANG, Xu-Liang BU, He-Lin YU, Peng LI, Hong-Yu OU, Ying HE, Fang-Di XU, Xiao-Yan HU, Xiao-Mei Zhu, Ping AO, Jun Xu

**Affiliations:** 1 Ministry of Education Key Laboratory of Systems Biomedicine, Shanghai Centre for Systems Biomedicine, Shanghai Jiao Tong University, Shanghai 200240, China; 2 State Key Laboratory of Microbial Metabolism and School of Life Sciences and Biotechnology, Shanghai Jiao Tong University, Shanghai 200240, China; 3 Institute of Oceanology, Shanghai Jiao Tong University, Shanghai 200240, China

## Abstract

*Streptomyces xiamenensis* 318, a moderate halophile isolated from a mangrove sediment, produces the anti-fibrotic compound xiamenmycin. The whole genome sequence of strain 318 was obtained through long-read single-molecule real-time (SMRT) sequencing, high-throughput Illumina HiSeq and 454 pyrosequencing technologies. The assembled genome comprises a linear chromosome as a single contig of 5,961,401-bp, which is considerably smaller than other reported complete genomes of the genus *Streptomyces*. Based on the antiSMASH pipeline, a total of 21 gene clusters were predicted to be involved in secondary metabolism. The gene cluster responsible for the biosynthesis of xiamenmycin resides in a strain-specific 61,387-bp genomic island belonging to the left-arm region. A core metabolic network consisting of 104 reactions that supports xiamenmycin biosynthesis was constructed to illustrate the necessary precursors derived from the central metabolic pathway. In accordance with the finding of a putative ikarugamycin gene cluster in the genome, the targeted chemical profiling of polycyclic tetramate macrolactams (PTMs) resulted in the identification of ikarugamycin. A successful genome mining for bioactive molecules with different skeletons suggests that the naturally minimized genome of *S. xiamenensis* 318 could be used as a blueprint for constructing a chassis cell with versatile biosynthetic capabilities for the production of secondary metabolites.

*Streptomyces* species are high-GC Gram-positive bacteria that are well known for their ability to produce a wide variety of medically and agriculturally useful antibiotics and related compounds. This bacterial genus has been extensively studied regarding its complex secondary metabolism and morphological differentiation process to understand the biosynthesis of natural products and its mechanism of genetic regulation^1^. The biotechnological potential of *Streptomyces* species with an emphasis on their secondary metabolism has been fully investigated at the systems level with the advent of high-throughput genome sequencing techniques and up-to-date bioinformatics tools^2^.

The genus *Streptomyces* is one of the most highly sequenced, with over hundreds of draft assemblies in the GenBank database. The limited number of finished *Streptomyces* genomic sequences reflects the challenges in completing the whole genome sequence for this group of bacteria^3^. The entire genome sequences of *S. lividans* TK24 and *S. lividans* 66^4^,^5^ were recently released, approximately ten years after the announcement of the first complete *Streptomyces* genome of their close affiliate *S. coelicolor* A3(2). The genome of *S. leeuwenhoekii* that consist of a linear chromosome and two extrachromosomal replicons were assembled as single contigs recently^6^, demonstrating the feasibility of combining the Pacific Biosciences SMRT (PacBio) and Illumina MiSeq technologies to generate the whole genome sequence of a *Streptomyces* species.

The chromosomes of *Streptomyces* species are always linear^7^, and spontaneous deletions, amplifications and arrangements occur at the telomeres, leading to genetic instability of the chromosome^8^. The Actinoblast database of reciprocal BLASTP best hits between genomes of the model strain *S. coelicolor* A3(2) and more than 100 other actinobacterial genomes provides a good reference for understanding the acquisition and the roles of actinobacteria-specific genes and processes during evolution^9^. A clearer view regarding the formation of the phylogenetic branch that led to *Streptomyces* could be achieved by assessing molecular signatures, such as clade-specific In/Dels^10^. Moreover, a naturally minimized *Streptomyces* genome, for example, a linear chromosome in *S. albus* J1074 that is 6.84 Mb in size^11^, would prove advantageous for defining the core genome of the *Streptomyces* genus. Furthermore, the reduced genome could be used as a good reference for the identification of niche-specific genes that lead to environmental adaptation, as demonstrated in the comparative genomics study of the sponge-associated *Streptomyces* spp. closely related to *S. albus* J1074^12^.

*S. xiamenensis* 318 was first isolated from the mangrove soil as a novel species of the *Streptomyces* genus^13^ and produces an anti-fibrotic drug lead, xiamenmycin^14^,^15^,^16^. The biosynthetic pathway of xiamenmycin was elucidated^17^, indicating a close connection between its biosynthesis and primary metabolism. In this study, we reported the whole genome sequence of *S. xiamenensis* 318 in a 5.96 Mb single contig, which is considerably smaller than other reported complete genomes of *Streptomyces* species. A central metabolic network that supports the biosynthesis of xiamenmycin was constructed, and comparative genomics analysis suggested that this naturally streamlined genome could be used as a blueprint for constructing a chassis cell for the biosynthesis of natural products.

## Results

### Genome structure

**Figure 1:**
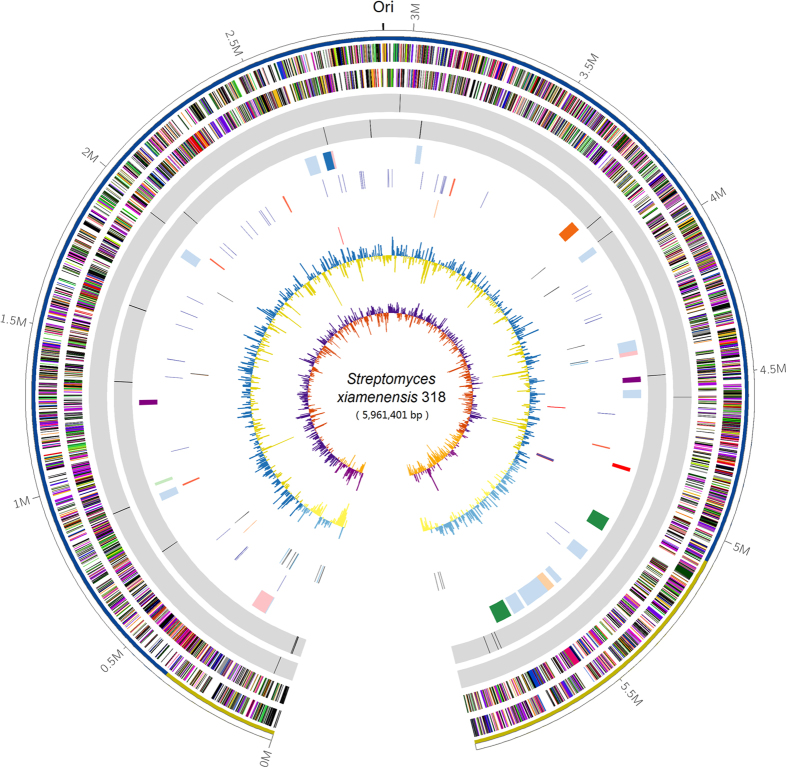
Schematic representation of the *S. xiamenensis* 318 linear chromosome. The outer scale is numbered in intervals of 0.5 Mb from the left to the right ends. Circle 1 in the solid line shows the core (blue) and arms (yellow); in Circles 2 and 3 (forward and reverse strands), the predicted protein coding regions are colored according to the COG function categories; Circles 4 and 5 show the *Dra*I and *Ase*I sites; in Circle 6, the distribution of secondary metabolic gene clusters are colored to show different product groups, and the pink blocks indicate a region that could be recognized as a genomic island; in Circle 7, the distribution of tRNAs (light blue) and rRNA operons (red) is shown; Circle 8 shows recombinases, transposes and integrases; Circle 9 shows the CRISPR array (pink) and Cas proteins (blue); Circle 10 indicates the GC content; Circle 11 presents the GC bias. Ori, origin of replication.

The size of the genome sequence assembly in a single contig is 5,961,401 bp, suggest that *S. xiamenensis* 318 has the most compact genome of the publicly available complete genomes of *Streptomyces* species in GenBank. The general features of the chromosome of *S. xiamenensis* 318 are summarized in Table 1 and Fig. 1. The predicted 5,491 protein coding sequences (CDSs) in *S. xiamenensis* 318 could be classified into 22 COG categories. The majority of the CDSs could be annotated with a putative function (35.3%) and an unknown function that has a homologue in GenBank (52.7%).

Although there is no discernible GC skew around *ori*, the genes related to DNA replication, including *oriC*, *dnaA*, and *dnaN*, were found to be clustered in the region at 2,911,121 to 2,911,310 bp (Fig. 1). Genes encoding the conserved terminal protein (Tpg, SXIM_53150) and telomere-associated protein (Tap, SXIM_53140) were identified in the right-arm region of the genome. The last one of the CDSs (SXIM_54840) shows high similarity to the telomere-proximal helicase-like gene *ttrA* that was previously identified in the TIR of the *S. lividans* chromosome and the right arm of SLP2^18^. Although we were not able to identify terminal repeat sequences in the sequencing attempts, the topology of the chromosome was proven to be linear, and this finding is supported by the physical maps generated by both *Dra*I and *Ase*I digestions (Supplementary Table S1) and Pulse-Field Gel Electrophoresis (PFGE; Supplementary Figure S1).

Twelve *Ase*I fragments larger than 70.4 kb could be determined by PFGE, resulting in a total chromosome size of approximately 5876.5 kb, whereas only six small fragments with a total size of 84.9 kb could not be detected in the chosen PFGE condition for separating large DNA fragments. Moreover, five *Dra*I sites were predicted based on the genome sequence, and these were all confirmed by sequencing of the small PCR products that were amplified with primers targeting the flanking regions of each *Dra*I site (PCR primer information is listed in Supplementary Table S2). Six *Dra*I fragments could be determined by PFGE, giving a total chromosome size of approximately 5961.4 kb, which matches the genome sizes calculated by *Ase*I mapping and by whole genome sequencing.

### *S. xiamenensis* 318 has a compact genome

A synteny analysis of the *S. xiamenensis* 318 genome compared with other representative complete genomes in the genus *Streptomyces* showed that *S. xiamenensis* 318 has a conserved core region of 4.8 Mb and two short arm regions of 0.36 and 0.8 Mb (Supplementary Figure S2).

**Figure 2:**
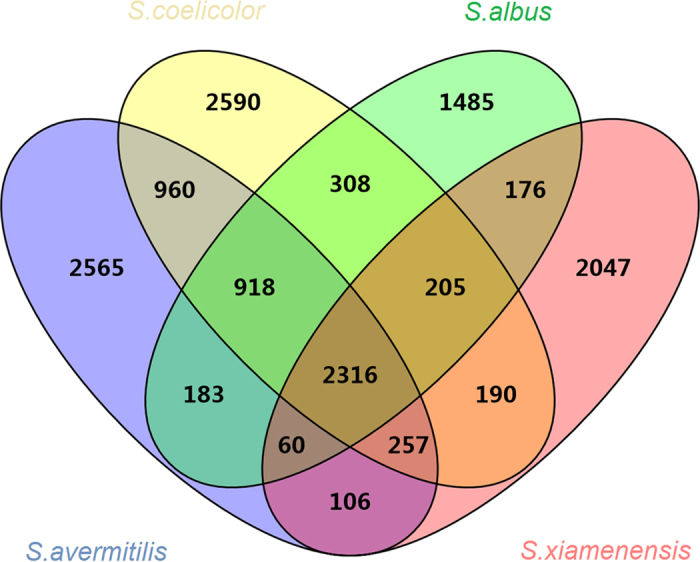
OrthoMCL analysis of *S. xiamenensis* 318, *S. albus* J1074, *S. avermitilis* MA-4680 and *S. coelicolor* A3(2). The number of shared and unique clusters is summarized as a Venn diagram, and the proteins were clustered using OrthoMCL parameters: e-value 1e-5; identity 50%; coverage 50% ; score 40; MCL Markov clustering inflation index 1.5.

Based on a OrthoMCL clustering analysis of *S. xiamenensis* 318, *S. albus* J1074, *S. avermitilis* MA-4680 and *S. coelicolor* A3(2) (Supplementary Table S3), the CDSs in *S. xiamenensis* 318 were clustered into 5,357 families, and 43.2% of these gene families were found to be commonly shared with the other three *Streptomyces* genomes (Fig. 2). The 2,047 unique gene families (38.2%) may be associated with the unique biological characteristics of *S. xiamenensis*. A paralogue analysis of each of the above-mentioned *Streptomyces* genomes is shown in Table 2 and Supplementary Table S4. The difference in the number of gene paralogues corresponds with the difference in the size of the genomes of these *Streptomyces* species. Fewer gene duplications may be one of the reasons explaining the further reduced genome size of *S. xiamenensis* 318.

A reciprocal best-hit BLAST analysis was performed between two naturally minimized chromosomes, namely *S. xiamenensis* 318 (5.96 Mb) and *S. albus* J1074 (6.8 Mb). The result shows that *S. xiamenensis* 318 has fewer genes in the majority of the COG groups except the gene group for carbohydrate transport and metabolism and the gene group for cell cycle control, cell division and chromosome partitioning (Supplementary Table S5). The genome size of *S. xiamenensis* 318 is 0.88 Mb smaller than that of the wildly-used *Streptomyces* host strain, *S. albus* J1074, corresponding to a balanced ribosomal capacity represented by five *rrn* operons in *S. xiamenensis* 318 instead of seven *rrn* operons in *S. albus* J1074.

Most *Streptomyces* species have at least six *rrn* operons. The positions of each of the *rrn* operons in six representative *Streptomyces* species were analysed (Supplementary Figure S3). It is interesting that one of the *rrn* operons, which is always inserted into a position between two conserved proteins, CDP-alcohol phosphatidyltransferase and phosphoenolpyruvate-dependent sugar phosphotransferase, was missing (Supplementary Figure S4) in *S. xiamenensis* 318, as confirmed through a re-sequencing of a PCR fragment that covered part of SXIM_03000 and SXIM_03010 and their intergenic region.

The numbers of tRNA genes in 16 complete *Streptomyces* genomes are summarized in Supplementary Table S6. In general, *Streptomyces* has 43 types of tRNA genes that yield a total number of 63 to 74 tRNAs. *S. xiamenensis* 318 presents less duplication in 11 types of tRNA genes. It is worth mentioning that the conserved pattern of the five tandem tRNA genes, which consisted of three tRNA-Glu genes and two tRNA-Gln genes, was not observed in *S. xiamenensis* 318 and *S. cattleya*. The linked tRNA gene locus only contains two tRNA-Glu genes and one tRNA-Gln gene in *S. xiamenensis* 318 (information on the PCR primers used for amplifying these tRNA-Glu/Gln genes and subsequent sequencing confirmation is presented listed in Supplementary Table S2). Moreover, the only tRNA-Asn gene in all *Streptomyces* species is always duplicated as a linked gene locus, but there is only one copy of this tRNA-Asn gene in *S. xiamenensis* 318. The reduction of the number of *rrn* operons to five copies and the maintenance of the total number of tRNA genes equal to 55 are hypothesized to be in accordance with the streamlined genome size of *S. xiamenensis* 318.

### Genomic island and genome plasticity

The restriction-modification system and CRISPR-Cas are well known prokaryotic defence systems. SXIM_28320 and SXIM_28330 encodes a putative McrBC subunit, indicating that a methylation-dependent restriction endonuclease system together with a typical type I, II, and III restriction and modification system may exist in *S. xiamenensis* 318 (Supplementary Table S7). Five CRISPR arrays were identified using CRISPR Finder (Supplementary Table S8).

**Figure 3:**
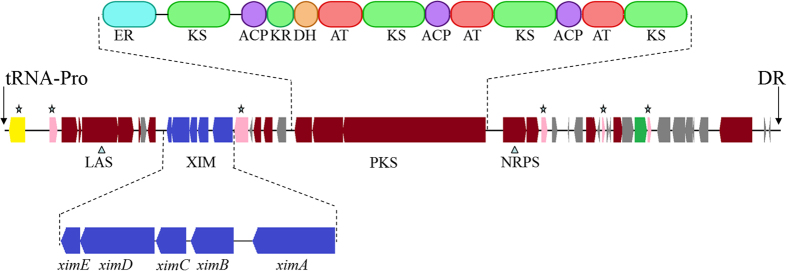
Structure of the genomic island GI-1. A 61,387-bp genomic island (GI-1) ranging from 200,207 to 261,593 in the left arm of the *S. xiamenensis* chromosome carries 46 protein-coding genes (SXIM_01760 to SXIM_02200). LAS: putative lantibiotic biosynthesis genes, including lantibiotic dehydratase (SXIM_01800) and lanthionine synthetase C family protein (SXIM_01810); XIM: xiamenmycin biosynthesis genes *ximA-E* (SXIM_01890 to SXIM_01850); PKS: polyketide synthase (SXIM_01950, SXIM_01960 and SXIM_01970). KS (keto synthase), AT (acyltransferase), DH (dehydratase), KR (ketoreductase), ACP (acyl-carrier protein). NRPS: non-ribosomal peptide synthase (SXIM_01980). DR: 43-bp direct repeat of the 3′-end of the tRNA-Pro gene (SXIM_t01). Pink bars with a star symbol: transposases (SXIM_01770, SXIM_01900, SXIM_02000, SXIM_02060 and SXIM_020120). Yellow bar with a star symbol: integrase (SXIM_01760).

26 genes were annotated as transposases and integrases. Six of these are clustered in the *S. xiamenensis* 318 strain-specific 61,387-bp genomic island (GI-1) ranging from 200,207 to 261,593 in the left arm of the chromosome, indicating hot spots for the integration of prophages and transposons. The GI-1 has a G + C content of 55%, which is abnormally lower than the average G + C content of 72% of the total genome, with two defined boundaries consisting of 43-bp direct repeats of the 3’-end of tRNA-Pro-GGG (SXIM_t01) gene. Remarkably, the gene cluster that is responsible for xiamenmycin biosynthesis is located in GI-1 (Fig. 3). In addition, two other genomic islands with a relatively low G + C content were found: GI-2 (SXIM_24830 - SXIM_24850, G + C % = 63%) and GI-3 (SXIM_41070- SXIM_41210, G + C % = 64%). These two islands are located within the core region and are adjacent to putative restriction-modification genes.

*S. xiamenensis* 318 (=MCCC 1A01550 = DSM 41903 = CGMCC 4.3534) was first isolated from a mangrove sediment^13^. Phylogenetically closely related strains with high identity (>98%) in the 16S rRNA gene sequence (Supplementary Table S9) have been reported, and these include *Streptomyces* sp. ZJ306 (16S rRNA Gene, access No. GI: 588481308) isolated from a sediment sample collected near the Pearl River estuary, South China Sea^19^, *Streptomyces* sp. AA0539 (16S rRNA Gene Locus extracted from genome sequence data, WGS, access No. GI: 485055197) isolated from a marine sediment collected near the shore in the Yellow Sea, China^20^, and *Streptomyces* sp. NRRL F-2890 (16S rRNA Gene Locus extracted from genome sequence data, WGS, access No. GI: 664205991). The draft genome sequences of *Streptomyces* sp. AA0539 and *Streptomyces* sp. NRRL F-2890 have been released^20^, the genome-to-genome distances (GGDH) between these three genomes were evaluated^21^ (Supplementary Table S9). Although *Streptomyces* sp. NRRL F-2890 and *S. xiamenensis* share very high similarity at the whole-genome level (GGDH value 96.6% ± 0.95), the *xim* gene cluster together with the 61.4-kb genomic island GI only exists in *S. xiamenensis* 318.

### Secondary metabolism

The potential of *S. xiamenensis* 318 to produce secondary metabolites was evaluated using the antiSMASH server^22^. Twenty-one gene clusters assumed to be involved in secondary metabolism could be catalogued into several major groups, including polyketide (PKS) and non-ribosomal polyketide (NRPS) (9), terpenoid (5), siderophore (2), lantipeptide (2), butyrolactone (2) and ectoine (1) (Supplementary Table S10). The total length of these gene clusters occupied 13.7% of the genome, specifically total lengths of 463.3 kb in the arms region and 352.8 kb in the core region.

The DNA fragment (cluster 1, Supplementary Table S10) located in the GI-1 is the only region in the left arm that is predicted to be involved in the biosynthesis of secondary metabolites. In addition to the characterized xiamenmycin biosynthesis gene cluster, the genes likely involved in lantipeptide, polyketide and non-ribosomal polyketide synthesis were identified (Fig. 3). One of the largest gene clusters (104.6 kb, clusters 19, Supplementary Table S10) located in the right-arm region (5,502,368 – 5,606,979) of the chromosome consists of terpene cyclase and about 20 kb multi-modular NRPS genes; The gene flanking to this NRPS was predicted to belong to the Streptomyces Antibiotic Regulatory Protein (SARP) family, which might control the expression of the downstream NRPS gene clusters.

**Figure 4:**
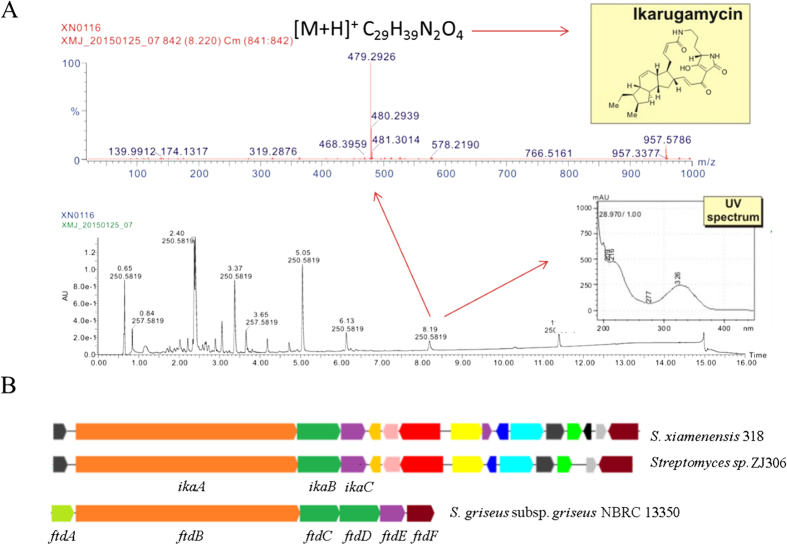
Chemical characterization of polycyclic tetramate macrolactams (PTMs) in the extract of *S. xiamenensis* 318 broth. (**A**). UPLC-UV profiling at 254 nm. The HR-MS spectrum, the UV profile of one of the PTM peaks and the structure of ikarugamycin are shown. (**B**). The genes involved in PTM biosynthesis in *S. xiamenensis* 318 were aligned to the reported ikarugamycin biosynthesis gene cluster from *Streptomyces* sp. ZJ306 and its counterpart in *S. griseus* subsp. *griseus* NBRC 13350.

A gene cluster (cluster 11, Supplementary Table S10) that annotated as hybridized type I PKS and NRPS spanning 58.4 kb showed high sequence identity to the 36.8 kb ikarugamycin biosynthesis gene cluster (Access No. GI: 746616581). A targeted chemical profiling of PTMs resulted in the identification of ikarugamycin, and this finding was confirmed by comparison of the retention time and accurate mass with an authentic standard sample by UPLC-QTOF-MS (Fig. 4). This result demonstrates the biosynthetic potential of *S. xiamenensis* 318 for the production of natural products other than xiamenmycin.

### Morphological and metabolic regulation network.

The *bld* regulatory cascade plays an important role in the morphological differentiation and antibiotic production in *Streptomyces* species^9^. Putative *bldA* (UUA-reading tRNA-Leu, 2,040,904-2,040,823), *bldB* (SXIM_44830), *bldD* (SXIM_03450), *bldG* (SXIM_41700) and *bldB* (SXIM_3710) were identified in *S. xiamenensis* 318. Identification of the A-factor biosynthesis protein AfsA (SXIM_17800) and homologues of the regulatory protein AfsR (SXIM_33050 and SXIM_42730) suggests that the gamma-butyrolactone signal transduction network may be used in *S. xiamenensis* 318.

TTA codon is comparatively rare in the GC-rich genomes of *Streptomyces*. There are 119 CDSs containing the rare codon TTA that may be regulated by *bldA* (Supplementary Table S11). According to the annotation, 27 transcriptional regulators, including AfsR (SXIM_33050) and a putative ECF (extracytoplasmic function) sigma factor (SXIM_34510), may coordinate with *bldA* in the complex regulatory network. Interestingly, TTA-containing *ximC* genes (SXIM_01870) and a putative lantibiotic dehydratase gene (SXIM_01800) were found to be located in the same genomic island, GI-1.

### Essential metabolic network for the biosynthesis of xiamenmycin

**Figure 5:**
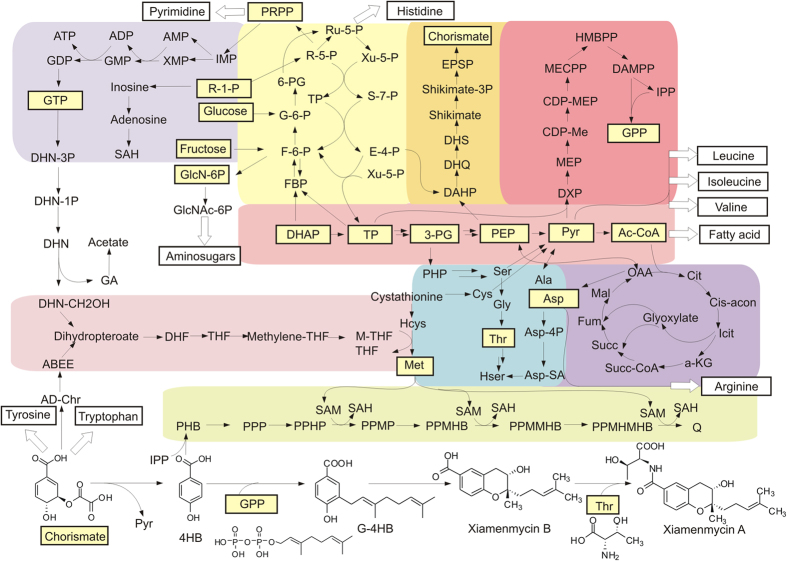
Essential metabolic network of *S. xiamenensis* supporting xiamenmycin biosynthesis. Four steps are involved in the biosynthesis of xiamenmycin (bottom of the panel), and the direct precursors, which are biosynthesized from shikimate biosynthesis (orange box), the non-mevalonate pathway (red box) and threonine biosynthesis (blue box), are mapped. The central carbon metabolism for *S. xiamenensis* 318 is illustrated in the schematic diagram by arrows and includes glycolysis/gluconeogenesis, the citric acid cycle (purple box), the pentose phosphate pathway (yellow box), the glyoxylate shunt (purple box), ubiquinone biosynthesis (green box), amino acid (i.e., glycine, serine, threonine, methionine, aspartate, alanine) metabolism (blue box), purine (GTP) synthesis (light purple box) and one carbon pool (pink box). The other multiple reactions involved in the synthesis of pyrimidine, fatty acid, histidine, tyrosine and tryptophan biosynthesis, leucine, isoleucine, valine, and arginine synthesis, and the synthesis of amino sugars are summarized as one step, which is marked by hollow arrows. The details of the reactions and metabolites are presented in Supplementary Table S12.

The core metabolic network of *Streptomyces* was constructed based on the comparison of genome information among *S. xiamenensis* 318, *S. albus* J1074 and *S. coelicolor* A3(2). The 82 sets of pathways that were found to be shared by the three *Streptomyces* genomes are listed in Supplementary Table S12. The reconstructed metabolic network consisting of 104 reactions (Fig. 5) that supports xiamenmycin biosynthesis was selected manually from the mutual pathways, which include glycolysis/gluconeogenesis, citrate cycle (TCA), pentose phosphate pathway (PPP), glyoxylate shunt, ubiquinone biosynthesis, purine metabolism, glycine, serine and threonine metabolism, methionine metabolism, and one carbon pool metabolism. The xiamenmycin-related pathways, i.e., shikimate biosynthesis, non-mevalonate pathway and threonine biosynthesis, may provide the direct precursors, i.e., 4-hyrobenzoic acid (4-HB), geranyl diphosphate (GPP) and threonine (Thr), respectively. The key metabolites erythrose-4-phosphate (E-4-P) from PPP, phosphoenolpyruvate (PEP) from glycolysis and pyruvate from TCA are the preferential sources for the biosynthesis of the precursors. Therefore, xiamenmycin biosynthesis is closely connected to central metabolism, as depicted in the constructed essential metabolic network (Fig. 5).

Chorismate and IPP are always used as building blocks for ubiquinone biosynthesis, as is evident in the metabolic pathways of *S. albus*, *S. coelicolor* and *S. xiamenensis*. Moreover, the precursors chorismate and isopentenyl pyrophosphate (IPP) are also used to synthesize xiamenmycin, which is a unique feature of *S. xiamenensis*. The transcriptional levels of the *xim* genes and the *ubi* genes that encode chorismate lyase and prenyltransferase were evaluated by RT-PCR (Supplementary Figure S5). The concordance in the changing pattern of the expression of the *xim* and *ubi* genes suggests a close relationship between the biosynthesis pathways. The metabolic network (Fig. 5) sheds light on the theoretical checking point responsible for the redirection of the metabolic flux toward either xiamenmycin or ubiquinone, which includes the splitting of chorismate to generate 4-HB as the starting unit.

## Discussion

### Potential as chassis cells for natural-product production

Systematics-guided bioprospecting in marine microbes has become an efficient approach for drug lead discovery^23^. As a moderate halophile with a naturally reduced genome, *S. xiamenensis* 318 is hypothesized to serve as a potential host for the heterologous expression of the gene clusters of interest identified from the metagenomic library constructed using marine-related samples.

Although there are four types of putative restriction enzymes, no obvious restriction to foreign DNA was found in transformation and conjugation. A 52-bp conservative *attB* site (2,970,893-2,970,945) close to the *ori* could be recognized by the phiC-31 phage recombinase, and the popular vector pSET152 was successfully integrated at *attB* in the chromosome of *S. xiamenensis* 318 (Supplementary Figure S6). This observation supports the hypothesis of using *S. xiamenensis* 318 as a surrogate host for the site-specific integration of foreign genes at the *attB* site.

As predicted by the antiSMASH pipeline, at least 13.7% of the compact genome of *S. xiamenensis* 318 still encodes secondary metabolites. The gene cluster responsible for the biosynthesis of PTM compounds contains an unusual hybrid polyketide synthase-nonribosomal peptide synthetase that is widely distributed in the genomes of phylogenetically diverse strains^24^; however, the activation of this type of cryptic gene cluster requires a synthetic biology strategy^25^. The PTM-type compound ikarugamycin was the main product identified in a chemical investigation of *Streptomyces* sp. ZJ306, which could be taxonomically defined as *S. xiamenensis* with 99% identity in the 16S rRNA gene sequence^19^. The feasibility of using this strain to express PKS or NRPS for the production of valuable natural products was shown by the detection of PTM compounds in the culture broth of *S. xiamenensis* 318 (Fig. 4).

### Further genome optimization

Based on a comparative genomic study of five *Streptomyces* species, a core genome of 3,096 orthologous families has been proposed^26^. A recent comparative genomic analysis of 17 *Streptomyces* species revealed that 34,592 ortholog clusters constituted the pan-genome of these *Streptomyces* species, including 2,018 in the core genome^27^. As shown in Supplementary Figure 7 and Supplementary Table S13, the inclusion of the genome of *S. xiamenensis* 318 in the dataset used for comparison further reduced the size of the core genome, even in the presence of *S. albus* J1074. This result suggests that the naturally reduced genome composition of *S. xiamenensis* 318 helps define the core genome of *Streptomyces* species.

In total, 352.8-kb gene clusters encoding putative endogenous secondary metabolites are sparsely distributed in the core regions in the linear chromosome of *S. xiamenensis* 318. With a size of 5.96 Mb, *S. xiamenensis* 318 might be one of the candidate strains for conducting further genome optimization with the aim of maintaining the core genome of *Streptomyces* species. The linear chromosomes and potentially linear chromosomes are generally larger than 7 Mb in size, whereas many circular chromosomes in Actinomycetales are smaller than 6 Mb^28^. The removal of a large DNA fragment from the linear chromosome of *S. xiamenensis* 318 may result in chromosomal circularization, as shown in the model *Streptomyces* species *S. coelicolor* A3(2)^29^. Whether the size of the 5.96-Mb genome of *S. xiamenensis* 318 represents the minimal requirement for a linear chromosome in *Streptomyce*s species is an open question.

All of the five genes that are responsible for xiamenmycin biosynthesis (*ximA-E*) in *S. xiamenensis* 318 have a high sequence similarity with their counterparts in *S. himastatinicus* ATCC 53653^17^. Excluding the homologues in *S. himastatinicus* ATCC 53653, the sequence similarities of the five *xim* genes obtained through a BlastP search against GenBank were less than 30%. There are two TTA codons in *ximC* gene, it is reasonable to infer that xiamenmycin biosynthesis could be controlled by regulating the expression level of the chorismate lyase (XimC) through the *bldA* regulatory cascade, which supposed to be coupled with morphological differentiation. The genes for xiamenmycin biosynthesis in *S. xiamenensis* 318 were hypothesized to be obtained by horizontal gene transfer and only exists in the strain-specific genomic island in *S. xiamenensis* 318, the true biological function of xiamenmycin in *S. xiamenensis* 318 should be considered in the background of environmental adaptation.

### Metabolic network

The analysis of the metabolic basis for xiamenmycin biosynthesis from a global point of view is helpful for the development of a cell factory to produce xiamenmycin. Reconstruction of a high-quality metabolic model enables the identification of gene overexpression targets for enhanced antibiotic production in *Streptomyces coelicolor* A3(2)^30^. The genome-scale metabolic flux maps of *S. lividans* TK24 growing under various physiological conditions have been obtained^31^. Because the xiamenmycin gene cluster can be expressed heterologously in *S. lividans*^17^, it is convenient to rapidly evaluate the flux distribution by adopting the metabolic model developed in the model *S. lividans* strain.

Considering only the central carbon metabolic pathway, the maximum carbon efficiency of xiamenmycin production in an engineered *S. lividans* has been estimated to be 20.0% on GYM media (glucose) and 27.0% on GYM (glucose) media supplemented with glycerol as an additional carbon source^32^. With a complete genome sequence at hand, it is possible to build metabolic models of the natural xiamenmycin-producing *S. xiamenensis* strain 318.

In this study, the complete genome sequence of *S. xiamenensis* 318 is presented as one of the most streamlined linear chromosomes found in the genus *Streptomyces*. Deciphering the genomic information will pave the road for the rational and systematic development of the *S. xiamenensis* 318 strain for the production of the promising anti-fibrotic drug lead xiamenmycin. Moreover, successful genome mining for bioactive molecules with different skeleton suggests that the naturally minimized genome of *S. xiamenensis* 318 could be used as a blueprint for constructing a chassis cell with versatile capabilities for the biosynthesis of secondary metabolites.

## Materials and Methods

### Genome sequencing and assembly

Genomic DNA was first sequenced using next-generation sequencing platforms. Pyrosequencing (454, Life Sciences) was performed by Majorbio Biotech (Shanghai, China), and Illumina pair-end (2*90 bp, 5-kb insert size) sequencing was performed by Genergy Bio (Shanghai, China). To achieve better sequence coverage and genome assembly, single-molecule real-time (SMRT) sequencing (Pacific Biosystems RS) was conducted at the Wuhan Institute of Biotechnology (Wuhan, China).

PacBio RS (long) reads were cleaned up with subreads in the SMRT portal, and only clean reads were included in the subsequent analyses. For assembly of the SMRT sequencing reads, the longest reads were first utilized as seeds to recruit all other short reads for the construction of highly accurate preassembled reads through a consensus procedure with HGAP^33^. Thereafter, the preassembled reads were constructed by aligning all of the reads to each of the seed reads using BLASR^34^. After the preassembly step, the resulting preassembled reads typically had read accuracies above 99%. Celera Assembler^35^ was then used to assemble all of the clean reads to the preassembly, and a new multi-read consensus algorithm Quiver (http://www.pacbiodevnet.com/quiver) was applied to generate the best consensus sequence as the final genome sequence result. Low-coverage contigs were removed from the final assembly, and Minimus2^36^ was used to connect all of the high-coverage contigs to ensure the proper connections between contigs. The short clean reads obtained from 454 and Illumina were used to correct the SMRT assembly. In this step, the short reads were mapped to the SMRT assembly with bwa and then sorted with SAMtools^37^, and the nucleotides of the assembly were corrected if they were different from the high-coverage short reads.

Initially, 240 Mb of sequence data was produced from the 454 sequencing platform, contained 625,536 reads with an average read length of 383.8 kb. The assembly consisted of 206 contigs of 5,909,744 bp with an average coverage of 40.6 X. After single-molecule real-time sequencing and data clean-up, 235.8 Mb of sequence data was obtained with an average subread length of 7.1 kb. During preassembly, 17,166 bp was taken as the cutoff for recruiting seed reads, and 150 Mb of seed reads was generated. After read correction, a 92-Mb preassembly with an average read length of 10.2 kb was obtained. The final assembly was composed of a chromosomal scaffold of 5,952,092 bp, with an estimated consensus concordance of 100% and an estimated sequencing depth of 290 X.

Then, 2.27 Gb from 22,768,408 trim reads generated using Illumina HiSeq were assembled using SOAPdenovo ver2.04 (http://soap.genomics.org.cn/soapdenovo.html)^38^. The N50 contig size was 471,947, and the largest contig was 1,097,189 nt, which gave 257 contigs with a total size of 5,963,650 bp (calculated coverage of 382 X). All of the contigs that were larger than 300 bp in the Illumina assembly showed both >99% identity and >99% coverage with the PacBio contigs, with the exception of one contig with a size of 38,094 bp. The sequence from 9,310 to 38,094 in this Illumina contig showed >99% identity to the sequence from 1 to 28,868 in the PacBio contigs. Thus, the extruding 9,309-bp sequence that showed no identity to the PacBio contigs was added to the almost complete genome of *S. xiamenensis* 318 in one contig of 5,961,401bp. The complete genome sequence has been deposited at GenBank under Accession No. GI: 921170702.

### Genome annotation and comparative analysis

OriC was predicted using the Ori-Finder (http://tubic.tju.edu.cn/Ori-Finder/) web server. Putative protein-coding sequences were predicted using Glimmer 3, which was trained with CDS from 10 completely sequenced *Streptomyces* genomes. Manual curation of all of the coding sequences was conducted by examining the database hits of BLAST 2.2.25 + against the NR database and the RAST server (http://rast.nmpdr.org/)^39^. tRNA and rRNA genes were predicted using the tRNAscan-SE 1.3.1 (http://lowelab.ucsc.edu/tRNAscan-SE/) and RNAmmer 1.2 (http://www.cbs.dtu.dk/services/RNAmmer/) web servers, respectively, and one predicted pseudo-tRNA (2,031,595-2,031,514) was manually assigned as the functional *bldA*. InterProScan 5 was used to confirm the domain assignments. GO term enrichment was performed using Blast2GO PRO 3.0. COG classification was performed using WebMGA online server (http://weizhong-lab.ucsd.edu/metagenomic-analysis/server/cog/). The prediction of the restriction and modification system was retrieved from the Restriction Enzyme Database (http://rebase.neb.com).

Genomic comparative analyses based on the Bidirectional Best Hit (BBH) methodology were performed using the following parameters: 30% query coverage and an e-value of 1e-5. The protein families were clustered with a local OrthoMCL 2.0.9 (http://orthomcl.org/orthomcl/) with the following cut-off values: identity, 50%; coverage, 50%; E-value, 1e-5; score, 40; MCL Markov clustering inflation index, 1.5. PanGP-windows-1.0.1 (http://pangp.big.ac.cn/download.html) was used in the pan-genome computation of *Streptomyces* species. Ortholog clusters were analysed through the open reading frame (ORF) contents of 17 *Streptomyces* genomes [GenBank access Nos. of the 17 genomes were reported by Kim J. *et al*. (2015)^26^] using OrthoMCL with the following parameters: E-value, 1e-10; score, 40; identity, 50; coverage, 50; MCL Markov clustering inflation index, 1.5. The 0/1 Matrix file for PanGP was generated using an in-house script based on the OrthoMCL results.

MUMmer 3.0 (http://mummer.sourceforge.net/) was used for *Streptomyces* chromosome sequence comparison. Secondary metabolite gene clusters were predicted by antiSMASH 2.0 (http://antismash.secondarymetabolites.org/) with additional manual curation^22^. CRISPR was predicted using the CRISPRfinder online program (http://crispr.u-psud.fr/Server/CRISPRfinder.php). CIRCOS 0.67-4 was used to generate the circular renditions of the genomic data and related annotations^40^. The core metabolic networks were constructed using the Model Seed server^41^. The GGDH value was obtained using the online Genome-to-Genome Distance Calculator (http://ggdc.dsmz.de/)^21^.

### Chemical profiling of the secondary metabolites

The crude extract for chemical profiling was prepared from the ethyl acetate extract of supernatant of the culture broth, and the cell debris was removed by filtration. UPLC-QTOF-MS was performed using a Waters ACQUITY UPLC system equipped with a Micromass Q-TOF Premier mass spectrometer (Waters MS Technologies, Manchester, UK). Chromatographic separations were performed on a 2.1 × 100-mm (1.7 μm) ACQUITY BEH C18 chromatography column. The column temperature was set to 45 °C, and the gradient eluting program was started from 5% B, changed to 30% B within 2 min, changed to 60% B within 4.5 min, changed to 100% B for another 7.5 min, and maintained at 100% B for 2 min (solvent A: aqueous solution of 0.1% formic acid; solvent B: ACN of 0.1% formic acid). The total flow rate was 0.40 mL/min. The eluate was directed to the mass spectrometer without splitting. Mass analysis was performed using a Q-TOF mass spectrometer equipped with an ESI source operating in the positive ion mode. The operating parameters were as follows: capillary voltage, 3.0 kV; sampling cone, 35 V; collision energy, 4 eV; source temperature, 100 °C; desolvation temperature, 300 °C; desolvation gas, 500 L/hr; scan range, m/z 50-1,000; scan time, 0.3 s; and interscan time, 0.02 s. The UV analysis was performed at a wavelength of 254 nm. Xiamenmycin A and ikarugamycin were detected using this method. The authentic standard sample of ikarugamycin was provided by Cayman Chemical (Ann Arbor, MI, USA). Xiamenmycin A and B were isolated and purified in our laboratory, and the purity obtained was 99%, as determined by HPLC.

### Genetic manipulation, RNA isolation and qRT-PCR assay

DNA extraction and manipulation in *S. xiamenensis* 318 were performed following the protocol described by Kieser *et al*.^42^. The integration vector pSET152 was introduced into *S. xiamenensis* 318 by conjugation between *E. coli* ET12567/pUZ8002 and *S. xiamenensis* 318. The exconjugants were selected as resistant colonies grown on SFM plate supplemented with 30 μg/ml apramycin. The integration of pSET152 was confirmed by sequencing a PCR fragment amplified by the primer set int_F and pirin_R (Supplementary Table S2), which covers the DNA fragments from both a chromosomal region and a vector region (see Supplementary Figure S6).

Cells in fermentation broth were collected at different time points. For total RNA extraction, the cells were rapidly cooling by pre-cold ethanol and harvested by centrifugation (12,000 rpm, 5 min, and 4 °C). The precipitate was then suspended in 1 mL of Trizol buffer (Molecular Research Centre) and immediately disrupted using glass beads and a BioSpec Mini-Beadbeater instrument (Mini-Beadbeater-24, Bartlesville, OK, USA). The total RNA was extracted with 200 μL of chloroform, precipitated with isopropanol, and suspended in DEPC water. The RNA was purified by the addition of RNase-free (Thermo Fisher Scientific) to completely remove the chromosomal DNA. Reverse transcription was achieved using the total RNA as the starting material and RevertAid H Minus reverse transcriptase according to the manufacturer’s procedure (Fermentas Thermo Scientific, Vilnius, Lithuania). The transcriptional levels of genes were determined by quantitative real-time RT-PCR (qRT-PCR) performed in a 7500 Fast Real-Time PCR system (Applied Biosystems, Foster City, CA, USA) with a One Step PrimeScript™ RT-PCR kit according to the manufacturer’s procedure (Takara, Dalian, China). The relative transcription levels of the target genes were normalized internally to the housekeeping gene *hrdB* (SXIM_45690) and quantified by the 2^− ∆∆CT^ method^43^. The sequences of the primer pairs are listed in Supplementary Table S2.

## Additional Information

**How to cite this article**: Min-Juan, X. *et al*. Deciphering the streamlined genome of *Streptomyces xiamenensis* 318 as the producer of the anti-fibrotic drug candidate xiamenmycin. *Sci. Rep*. **6**, 18977; doi: 10.1038/srep18977 (2016).

## Supplementary Material

Supplementary Information

Supplementary Table S3

Supplementary Table S4

Supplementary Table S5

Supplementary Table S6

Supplementary Table S9

Supplementary Table S11

Supplementary Table S12

Supplementary Table S13

## Figures and Tables

**Table 1:**
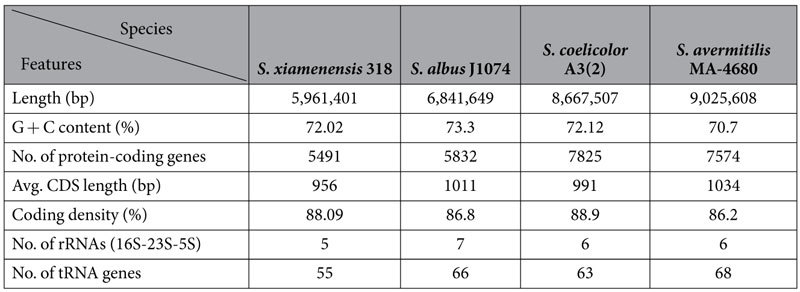
General features of the chromosomes of *S. xiamenensis* 318 and three other *Streptomyces* species.

**Table 2:**
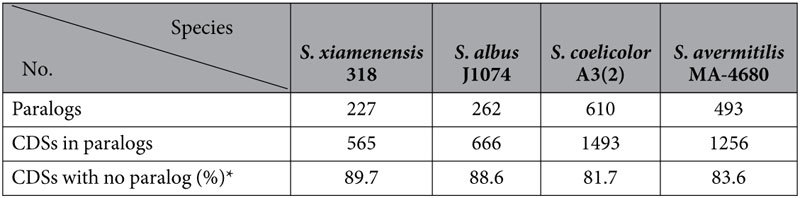
Gene paralogs in the genome of four *Streptomyces* species including *S. xiamenensis* 318.

*The ratio of CDSs without a paralog is given as the percentage of the total number of CDSs.
