# Supplementary material for: Deciphering the streamlined genome of Streptomyces xiamenensis 318 as the producer of the anti-fibrotic drug candidate xiamenmycin
Source: Sci Rep. 2016 Jan 8;6:18977. doi: 10.1038/srep18977 (PMC4705527; doi:10.1038/srep18977)
Supplement: Supplementary Information [file srep18977-s1.doc]

**Supplementary materials**

**Deciphering the streamlined genome of *Streptomyces xiamenensis* 318 as the producer of the anti-fibrotic drug candidate xiamenmycin**

Min-Juan XU^1^, Jia-Hua WANG^2, 3^, Xu-Liang BU^2, 3^, He-Lin YU^1^, Peng LI^2^, Hong-Yu OU^2^, Ying HE^2^, Fang-Di XU^2^, Xiao-Yan HU^2, 3^, Xiao-Mei Zhu^1^, Ping AO^1^ and Jun Xu^2, 3^*

^1^ Ministry of Education Key Laboratory of Systems Biomedicine, Shanghai Centre for Systems Biomedicine, Shanghai Jiao Tong University, Shanghai 200240, China

^2^ State Key Laboratory of Microbial Metabolism and School of Life Sciences and Biotechnology, Shanghai Jiao Tong University, Shanghai 200240, China

^3^ Institute of Oceanology, Shanghai Jiao Tong University, Shanghai 200240, China

*Correspondence and requests for materials should be addressed to J. X. (xujunn@sjtu.edu.cn)

**Subject areas**: genome reduction, *Streptomyces* genetics, natural product

**List of Supplementary materials**

1. Supplementary Fig. S1, Genome size estimation of *S. xiamenensis* 318 by pulse-field gel electrophoresis.
2. Supplementary Fig. S2, Synteny analysis of the *S. xiamenensis* 318 chromosome in comparison with other reported completely sequenced chromosomes in the genus *Streptomyces*.
3. Supplementary Fig. S3, Locations of the rRNA operons in the chromosomes of *S. xiamenensis* 318 and six other *Streptomyces* species.
4. Supplementary Fig. S4, Confirmation of the missing rRNA operon in *S. xiamenesis* 318.
5. Supplementary Fig. S5, Transcriptional levels of the *xim* genes and the *ubi* genes that encode chorismate lyase and prenyltransferase in *S. xiamenensis* 318 at different growth phases.
6. Supplementary Fig. S6, Confirmation of *attB* in the chromosome of *S. xiamenensis* 318 by pSET152 integration.
7. Supplementary Fig. S7, Pan-genome analysis of 18 *Streptomyces* species.

1. Supplementary Table S1, Predicted *Dra*I and *Ase*I sites in *S. xiamenensis* 318
2. Supplementary Table S2, Primers information for genomic studies (a) and Real-time PCR (b)
3. Supplementary Table S3, OrthoMCL clustering of *S. albus* J1074, *S. avermitilis*, *S. coelicolor* A3(2) and *S. xiamenensis* 318
4. Supplementary Table S4, Paralogue analysis of *S. albus* J1074, *S. avermitilis*, *S. coelicolor* A3(2) and *S. xiamenensis* 318
5. Supplementary Table S5, Best reciprocal hit BLAST analysis between *S. xiamenensis* 318 and *S. albus* J1074.
6. Supplementary Table S6, Numbers of tRNA genes in 16 complete *Streptomyces* genomes
7. Supplementary Table S7, Predicted restriction and modification system in *S. xiamenensis* 318
8. Supplementary Table S8, Predicted CRISPR (a) and Cas (b) system in *S. xiamenensis* 318
9. Supplementary Table S9, 16S rRNA gene sequences of four *S. xiamenensis* and evaluation of genome-to-genome distances (GGDH)
10. Supplementary Table S10, Predicted gene clusters involving in the biosynthesis of secondary metabolites
11. Supplementary Table S11, CDSs that contains TTA codon involving *bldA* regulation
12. Supplementary Table S12, List of the reactions in the reconstructed metabolic network that supports xiamenmycin biosynthesis
13. Supplementary Table S13, List of gene clusters from the genomes of 18 *Streptomyces* species used in pan-genome and core genome analysis.

| **Supplementary Table S1 | Predicted *DraI* and *AseI* sites in *S. xiamenensis* 318** | | | | | |
| --- | --- | --- | --- | --- | --- |
| DraI (TTT/AAA) | Fragment size | | AseI (AT/TAAT) | | Fragment size |
| 5 sites | bp |  | | 17 sites | bp |
|  | 67,028 |  | |  | 33,157 |
| 67,028 | 803,838 |  | | 33,157 | 3,908 |
| 870,866 | 1,102,397 |  | | 37,065 | 628,412 |
| 1,973,263 | 994,270 |  | | 665,477 | 22 |
| 2,967,533 | 1,610,334 |  | | 665,499 | 680,279 |
| 4,577,867 | 1,383,534 |  | | 1,345,778 | 294,076 |
|  |  |  | | 1,639,854 | 615 |
|  |  |  | | 1,640,469 | 411,202 |
|  |  |  | | 2,051,671 | 625,928 |
|  |  |  | | 2,677,599 | 178,609 |
|  |  |  | | 2,856,208 | 195,840 |
|  |  |  | | 3,052,048 | 785,752 |
|  |  |  | | 3,837,800 | 70,371 |
|  |  |  | | 3,908,171 | 584,819 |
|  |  |  | | 4,492,990 | 1,284,787 |
|  |  |  | | 5,777,777 | 12,670 |
|  |  |  | | 5,790,447 | 34,531 |
|  |  |  | | 5,824,978 | 136,423 |

| **Supplementary Table S2 | Primers information for genomic studies (a) and Real-time PCR (b)**  **(a)** | | | | | | | | | |  | |
| --- | --- | --- | --- | --- | --- | --- | --- | --- | --- | --- | --- |
|  | | Primer name | | Target | | Product size (bp) | | | Sequence (5'-3') | | |
|  | | **rRNA gene**  **confirmation** | |  | |  | | |  | | |
|  | | rr0_F | | rrna0, putative | | 1106 | | | CTTGCCCACGTAGTTGACCT | | |
|  | | rr0_R | |  | |  | | | GACTCCCTGCAGCATTGTCT | | |
|  | |  | |  | |  | | |  | | |
|  | | rr1_F | | rrna1 | | 2315 | | | CGGAACTTCCCAGTTCTGGT | | |
|  | | 23S_R | |  |  |  |  |  | CCCCTGCCGGGTATCACACA | | |
|  | | rr2_F | | rrna2 | | 2313 | | | AAAGTGGTGGACACGCCGGA | | |
|  | | 23S_R | |  |  |  |  |  | CCCCTGCCGGGTATCACACA | | |
|  | | rr3_F | | rrna3 | | 2315 | | | GGCCGGGGAAATCCCCTAAA | | |
|  | | 23S_R | |  |  |  |  |  | CCCCTGCCGGGTATCACACA | | |
|  | | rr4_F | | rrna4 | | 2215 | | | CGGCGGGGAAATCCTCTAAA | | |
|  | | 23S_R | |  |  |  |  |  | CCCCTGCCGGGTATCACACA | | |
|  | | rr5_F | | rrna5 | | 2315 | | | GTTTACGTCATCGCCGCAAC | | |
|  | | 23S_R | |  |  |  |  |  | CCCCTGCCGGGTATCACACA | | |
|  | |  | |  | |  | | |  | | |
|  | | Gln_tRNA_F | | Gln pattern | | 544 | | | TTTCATCCAGCGGGCGTACC | | |
|  | | Gln_tRNA_R | |  |  |  |  |  | ACGAACCCGTGCTGCTGAGC | | |
|  | |  | |  | |  | | |  | | |
| **DraI site**  **confirmation** | | | |  | |  | | |  | | |
|  | | DraI(1)_F | | DraI Site (57763) | | 501 | | | ATGAGCTTCCCCGGTCAGGA | | |
|  | | DraI(1)_R | |  |  |  |  |  | GCAACCTTGTCGGCTCCAGT | | |
|  | | DraI(2)_F | | DraI Site (861601) | | 501 | | | CCACGTGTTACTCACCCGTT | | |
|  | | DraI(2)_R | |  |  |  |  |  | TAGATCGAGTCGCTTGAGGC | | |
|  | | DraI(3)_F | | DraI Site (1963998) | | 801 | | | TCAAACTCTCCATGAATGCT | | |
|  | | DraI(3)_R | |  |  |  |  |  | CTGTTCGTCTCCGGACTGAT | | |
|  | | DraI(0)_F | | DraI Site (2000816), putative | | 477 | | | CGACCTTGCCCATGGTCACC | | |
|  | | DraI(0)_R | |  |  |  |  |  | TCCAGGCCGCAGACGGTGGC | | |
|  | | DraI(4)_F | | DraI Site (2958268) | | 475 | | | AAGTACGTCGACCAGCTCAC | | |
|  | | DraI(4)_R | |  |  |  |  |  | GATGACCGTGACCAAACGTT | | |
|  | | DraI(5)_F | | DraI Site (4568602) | | 458 | | | GCTCCTGGCGCGCTTGGCCC | | |
|  | | DraI(5)_R | |  |  |  |  |  | TCGGTCAGCGTCCGGTCACC | | |
|  | |  | |  | | | |  | |  | |
| **(b)** | | | |  | | | |  | |  | |
|  | Primer name | | Locus | | Description | | Sequence(5'-3') | | | |  |
|  | q_hrdB_F | | sxim_45690 | | *hrdB* | | GGTCGAGGTCATCAACAA | | | |  |
|  | q_hrdB_R | |  |  |  |  | CGTCATGTCCAGTTCCTT | | | |  |
|  | q_UbiC_F | | sxim_13770 | | *ubiC* | | TCGGTCAACCAGGTAGTC | | | |  |
|  | q_UbiC_R | |  |  |  |  | GCAGATACGTCTTGTAGCA | | | |  |
|  | q_4-HB_F | | sxim_13780 | | 4-hydroxybenzoate 3-monooxygenase | | CTTCTCCGAGCGTCTGAT | | | |  |
|  | q_4-HB_R | |  |  |  |  | TCTCCACTCTTTACAGTTCAC | | | |  |
|  | q_UbiA2_F | | sxim_13790 | | *ubiA* | | CACAAGTTCTGGCTGACC | | | |  |
|  | q_UbiA2_R | |  |  |  |  | CAGTCGTTGACGAGGTTG | | | |  |
|  | q_UbiE_F | | sxim_13800 | | *ubiE* | | GGATGCAGTTGCTGACCC | | | |  |
|  | q_UbiE_R | |  |  |  |  | CACATCGCTCACCGTCAC | | | |  |
|  | q_XimE_F | | sxim_01850 | | *ximE* | | CAAGCACTACTGGACAAC | | | |  |
|  | q_XimE_R | |  |  |  |  | GTTCGTGATCAACCCATC | | | |  |
|  | q_XimD_F | | sxim_01860 | | *ximD* | | TACTTGTCTGCCCGTTTC | | | |  |
|  | q_XimD_R | |  |  |  |  | CATCACCACCCACTGTTG | | | |  |
|  | q_XimC_F | | sxim_01870 | | *ximC* | | CAGTTCCGCACCGTTTAC | | | |  |
|  | q_XimC_R | |  |  |  |  | GTGTTCCTCGGGGCTTAT | | | |  |
|  | q_XimB_F | | sxim_01880 | | *ximB* | | GTCTTCGGCTGCTACATC | | | |  |
|  | q_XimB_R | |  |  |  |  | CGTAGATCGTGTCGTACC | | | |  |
|  | q_XimA_F | | sxim_01890 | | *ximA* | | GCCGAGGTACTGGGTAAC | | | |  |
|  | q_XimA_R | |  |  |  |  | CGTTCCGTAGGTGTTGAG | | | |  |

| **Supplementary Table S7 | Predicted Restriction and Modification (R&M) system in *S. xiamenensis* 318** | | | | | | | | |
| --- | --- | --- | --- | --- | --- | --- | --- | --- |
| R&M | Start | End | Strand | Putative function | Top hit in REBASE^╪^  (identity) | |  |  |
| Type I |  |  |  |  |  | | | |
| SXIM_24810 | 2673288 | 2671357 | - | DNA-methyltransferase subunit S | M.Asp2582ORF22435P,  gi|683979588 (55%) | | |  |
| SXIM_24830 | 2676290 | 2674278 | - | DNA-methyltransferase subunit M | M.Sal41398ORF3728P, gi|749178358 (72%) | |  |  |
|  |  |  |  |  |  | | | |
| Type II |  |  |  |  |  | | | |
| SXIM_18960 | 2058910 | 2057021 | - | adenine-specific DNA methylase | M.Spr10218ORF4437P, gi|924536561 (61%) |  |  | |
| SXIM_23980 | 2565976 | 2560304 | - | methylase subunits | Ssp150FBORF32760P, gi|747170153 (52%) |  | | |
| SXIM_24010 | 2570662 | 2574015 | + | adenine-specific DNA methyltransferase | Sno14899ORF25055P,  (58%) |  | | |
| SXIM_29640 | 3194225 | 3195250 | + | N-6 adenine-specific DNA methylase | M.Ssp150FBORF16915P, gi|747167959 (74%) |  | | |
| SXIM_43860 | 4669548 | 4670186 | + | methyltransferase | M.SspJS01ORF32935P, gi|674710734 (71%) |  | | |
|  |  |  |  |  |  | | | |
| Type III |  |  |  |  |  | | | |
| SXIM_41070 | 4375725 | 4377806 | + | methylation subunit | M.AspJ337ORFAP, gi|389596467 (42%) |  | | |
| SXIM_41080 | 4377803 | 4380229 | + | restriction endonuclease subunit | Ssp1322ORF11130P, gi|902962221 (47%) |  | | |
|  |  |  |  |  |  | | | |
| Type IV |  |  |  |  |  | | | |
| SXIM_28320 | 3056512 | 3055262 | - | McrC subunit | Sfu40593McrCP, gi|485097657 (43%) |  | | |
| SXIM_28330 | 3058689 | 3056536 | - | McrB subunit | Ssc87McrBP (51%) |  | | |

## ^╪^ REBASE, the Restriction Enzyme Database, summary of the putative R&M system in *Streptomyces xiamenensis* 318

(http://tools.neb.com/genomes/summary.php?genome_id=20297)

| **Supplementary Table S8 | Predicted CRISPR (a) and Cas system (b) in *S. xiamenensis* 318**  **(a)** | | | | |
| --- | --- | --- | --- | --- |
| CRISPR array | Start | End | Spacer | Length |
| 1 | 2619721 | 2619817 | 1 | 96 |
| 3 | 4646400 | 4646600 | 3 | 200 |
| 4 | 4961963 | 4963212 | 20 | 1249 |
| 5 | 4973326 | 4974207 | 14 | 881 |
| **(b)** |  |  |  |  |
| CRISPR associated proteins | Start | End | Strand | Annotation |
| SXIM46290 | 4963403 | 4966372 | + | CRISPR-associated helicase Cas3 |
| SXIM46300 | 4966591 | 4968369 | + | CRISPR-associated protein, Cse1 family |
| SXIM46310 | 4968366 | 4969046 | + | CRISPR-associated protein, Cse2 family |
| SXIM46320 | 4969092 | 4970273 | + | CRISPR-associated protein, Cse4 family |
| SXIM46330 | 4970273 | 4971118 | + | CRISPR-associated protein, Cas5e family |
| SXIM46340 | 4971123 | 4971923 | + | CRISPR-associated protein, Cse3 family |
| SXIM46350 | 4971928 | 4972986 | + | CRISPR-associated protein Cas1 |
| SXIM46360 | 4972983 | 4973261 | + | CRISPR-associated protein, Cas2 |

| **Supplementary Table S10 | Predicted gene clusters involving in the biosynthesis of secondary metabolites** | | | | | |
| --- | --- | --- | --- | --- | --- |
| Cluster No.^(*)^ | Product Type | start | end | Length  (bp) | Putative function^(╪)^ |
| *1* | *Lantipeptide-PKS* | *196117* | *262123* | *66,006* | *^╪^ Xiamenmycin biosynthetic gene cluster (consists of 5 genes )* |
| 2 | Siderophore | 842143 | 873779 | 31,636 | Desferrioxamine B biosynthetic gene cluster (60% of genes show similarity) |
| 3 | Ectoine | 910510 | 921175 | 10,665 | Ectoine biosynthetic gene cluster (75% of genes show similarity) |
| 4 | Terpene | 1252926 | 1274017 | 21,091 | Chlortetracycline biosynthetic gene cluster (5% of genes show similarity) |
| 5 | Butyrolactone | 1900897 | 1941604 | 40,707 | - |
| 6 | PKS/NRPS | 2569641 | 2618819 | 49,178 | - |
| 7 | PKS/NRPS | 2649340 | 2690629 | 41,289 | Simocyclinone biosynthetic gene cluster (13% of genes show similarity) |
| 8 | Butyrolactone | 3044395 | 3066389 | 21,994 | Methylenomycin biosynthetic gene cluster (9% of genes show similarity) |
| 9 | PKS | 3777683 | 3820231 | 42,548 | Lomaiviticin biosynthetic gene cluster (38% of genes show similarity) |
| 10 | Lantipeptide-linaridin | 3913176 | 3941259 | 28,083 | - |
| 11 | PKS/NRPS-linaridin | 4338936 | 4397384 | 58,448 | ^╪^ ikarugamycin biosynthetic gene cluster (25 genes show >92.7% amino acid sequence similarity) |
| 12 | Terpene | 4494723 | 4515865 | 21,142 | - |
| 13 | Lantipeptide-thiopeptide | 4549081 | 4583346 | 34,265 | - |
| 14 | Siderophore | 4879329 | 4893738 | 14,409 | - |
| *15* | *NRPS* | *5106208* | *5164444* | *58,236* | *Valinomycin biosynthetic gene cluster (16% of genes show similarity)* |
| *16* | *PKS/NRPS* | *5264516* | *5315551* | *51,035* | *Carotenoid biosynthetic gene cluster (18% of genes show similarity)* |
| *17* | *Terpene* | *5426796* | *5451770* | *24,974* | *Hopene biosynthetic gene cluster (30% of genes show similarity)* |
| *18* | *Terpene-PKS* | *5469251* | *5510381* | *41,130* | *Herboxidiene biosynthetic gene cluster (2% of genes show similarity)* |
| *19* | *Terpene-NRPS* | *5502368* | *5606979* | *104,611* | *Herboxidiene biosynthetic gene cluster (5% of genes show similarity)* |
| *20* | *PKS/NRPS* | *5620958* | *5672095* | *51,137* | *Lividomycin biosynthetic gene cluster (6% of genes show similarity)* |
| *21* | *NRPS* | *5686309* | *5752451* | *66,142* | *Kirromycin biosynthetic gene cluster (12% of genes show similarity)* |

^(*)^ gene clusters in italic font are located in the arms regions of the chromosome.

^(╪)^ product of xiamenmycin and ikarugamycin gene clusters detected in *S. xiamenensis* 318

**Supplementary Table S3, OrthoMCL clustering of *S. albus* J1074, *S. avermitilis*, *S. coelicolor* A3(2) and *S. xiamenensis* 318**

**(**Large supplementary datasets included as Excel file**)**

**Supplementary Table S4, Paralogue analysis of *S. albus* J1074, *S. avermitilis*, *S. coelicolor* A3(2) and *S. xiamenensis* 318**

**(**Large supplementary datasets included as Excel file**)**

**Supplementary Table S5, Best reciprocal hit BLAST analysis between *S. xiamenensis* 318 and *S. albus* J1074**

**(**Large supplementary datasets included as Excel file**)**

**Supplementary Table S6, Numbers of tRNA genes in 16 complete *Streptomyces* genomes**

**(**Large supplementary datasets included as Excel file**)**

**Supplementary Table S9, 16S rRNA gene sequences of four *S. xiamenensis* and evaluation of genome-to-genome distances (GGDH)**

**(**Large supplementary datasets included as Excel file**)**

**Supplementary Table S11, CDSs that contains TTA codon involving *bldA* regulation**

**(**Large supplementary datasets included as Excel file**)**

**Supplementary Table S12, List of the reactions in the reconstructed metabolic network that supports xiamenmycin biosynthesis**

**(**Large supplementary datasets included as Excel file**)**

**Supplementary Table S13, List of gene clusters from the genomes of 18 *Streptomyces* species used in pan-genome and core genome analysis**

**(**Large supplementary datasets included as Excel file**)**

**
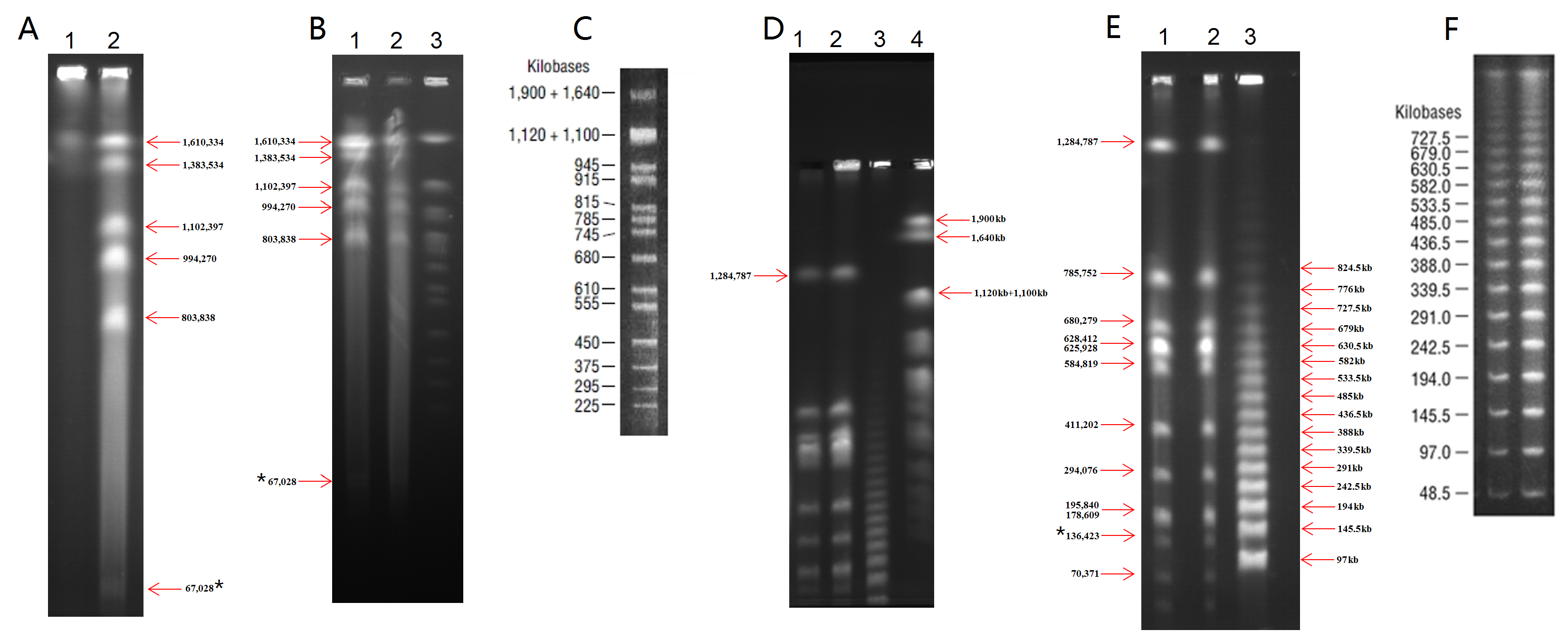
**

**Supplementary Fig. S1 | Genome size estimation of *S. xiamenensis* 318 by pulse-field gel electrophoresis.** The genomic DNA of *S. xiamenensis* 318 was embedded in agar plugs and treated by NDS and proteinase K, digested by either *Dra*I or *Ase*I, and fractionated by pulsed-field gel electrophoresis. Details of the *Dra*I or *Ase*I sites can be found in Supplementary Table S1. A: 1. Complete genomic DNA, 2. *Dra*I-digested genomic DNA. B: 1 and 2, *Dra*I-digested genomic DNA, 2. Yeast chromosome PFG marker. C: Product description of yeast chromosome PFG marker (Biolabs, #N0345S). D: 1 and 2, *Ase*I*-*digested genomic DNA, 3. Lambda-ladder PFG marker, 4. Yeast chromosome PFG marker. E: 1 and 2, *Ase*I*-*digested genomic DNA, 3. Lambda-ladder PFG marker. F: Lambda-ladder PFG marker (Biolabs, #N0340S).


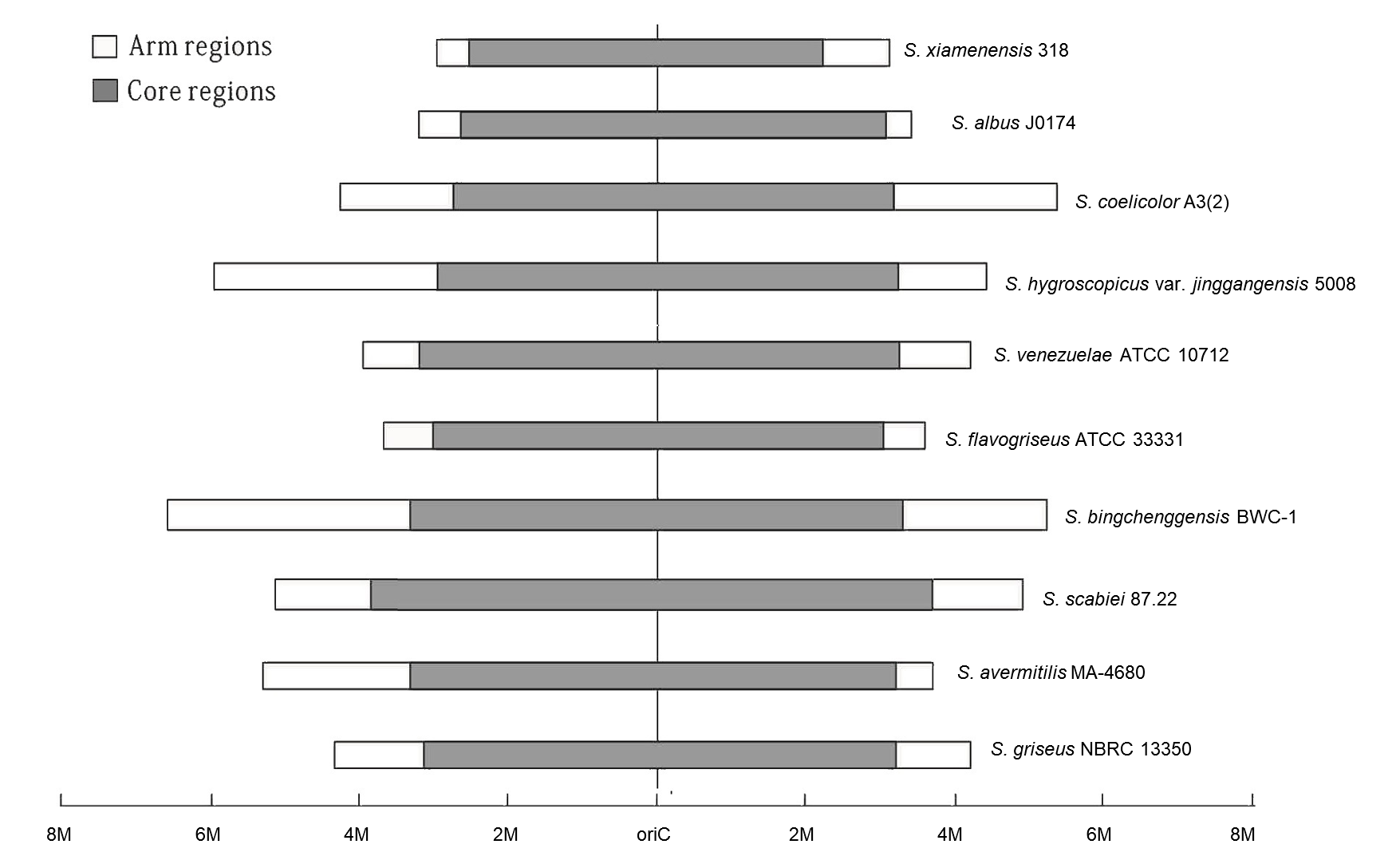


**Supplementary Fig. S2 | Synteny analysis of the *S. xiamenensis* 318 chromosome in comparison with other reported completely sequenced chromosomes in the genus *Streptomyces*.**


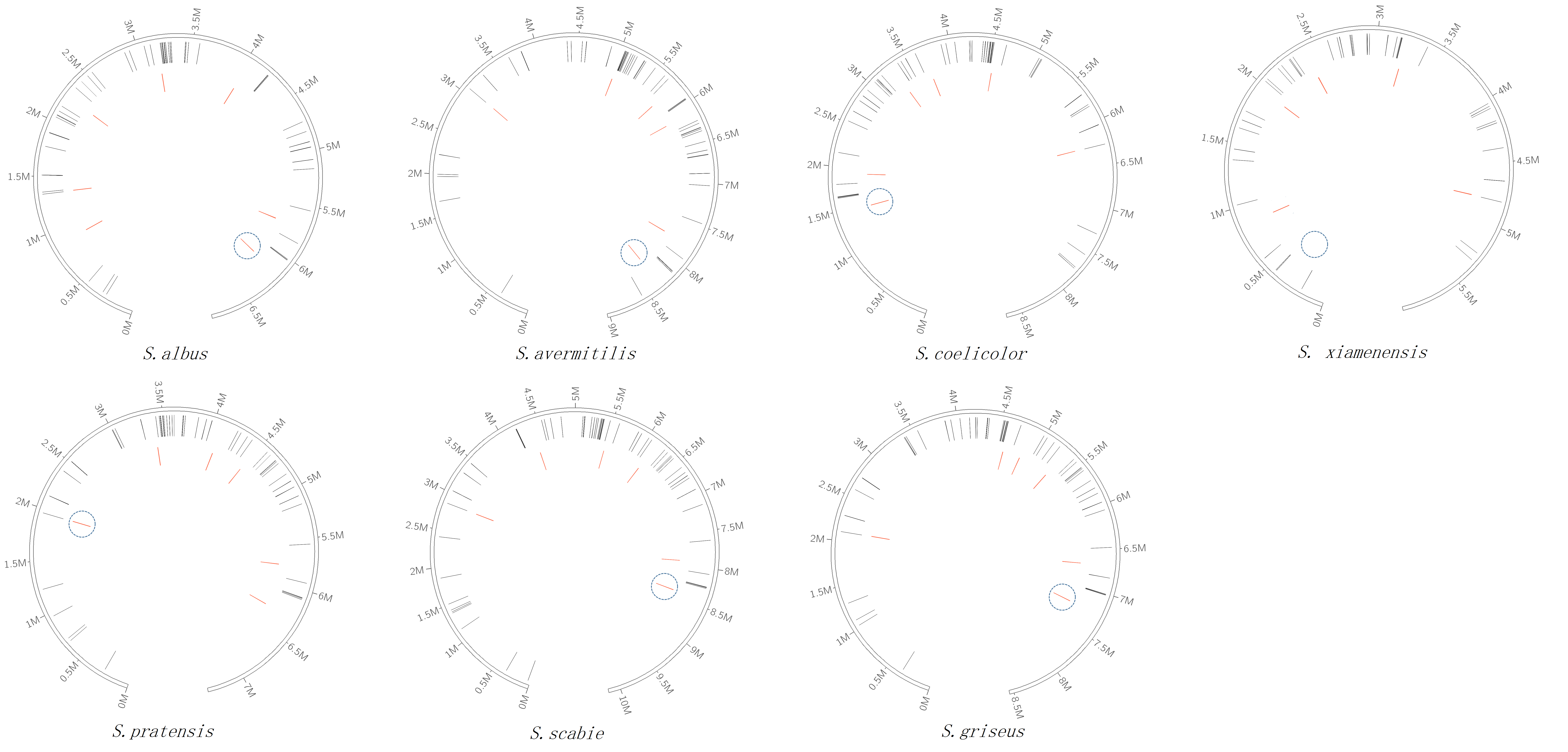


**Supplementary Fig. S3 | Locations of the rRNA operons in the chromosomes of *S. xiamenensis* 318 and six other *Streptomyces* species.** The positions of tRNA genes (black bar) and *rrn* operons (red bar) are labelled. The *rrn* operon circled with the dashed line has two highly conserved genes in the flanking regions in six representative *Streptomyces* species but is not found in *S. xiamenensis* 318.

**
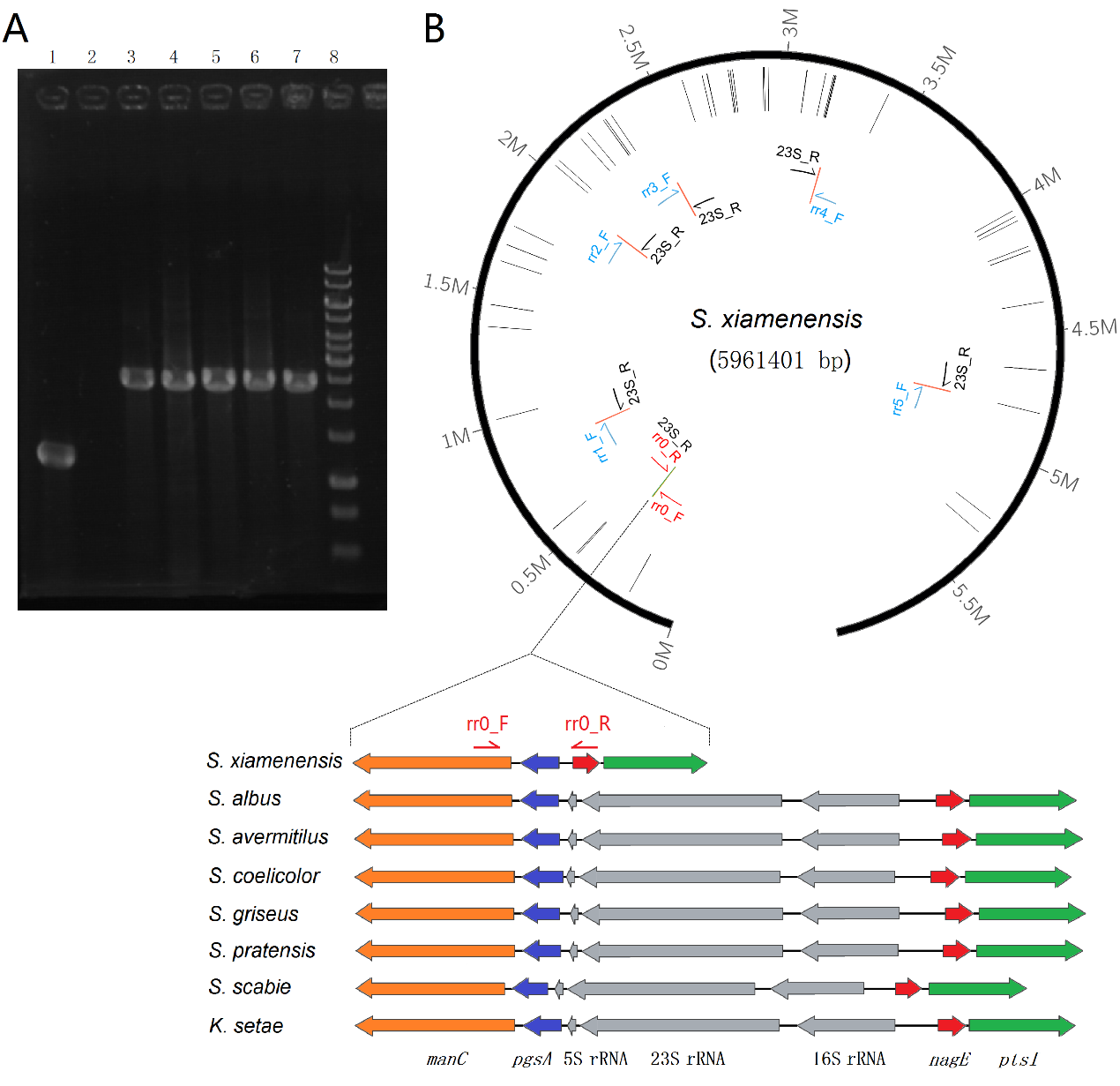
**

**Supplementary Fig. S4 | Confirmation of the missing rRNA operon in *S. xiamenesis* 318.** A: PCR amplification of *rrn* operons. Primer sets 1. rrn0_F/R, 2. rrn0_F/23S_R, 3. rr1_F/23S_R, 4. rrn2_F/23S_R, 5. rrn3_F/23S_R, 6. rrn4_F/23S_R, 7. rrn5_F/23S_R, 8. Thermo GeneRuler 1 kb DNA Ladder; B: Schematic diagram show the positions of the *rrn* operons. The putative *rrn*0 operon are located in two conserved proteins (CDP-alcohol phosphatidyltransferase and phosphoenolpyruvate-dependent sugar phosphotransferase ) in 6 *Streptomyces* species and *kitasatospora setae*, but was confirmed to be missing in *S. xiamenesis* 318**.**


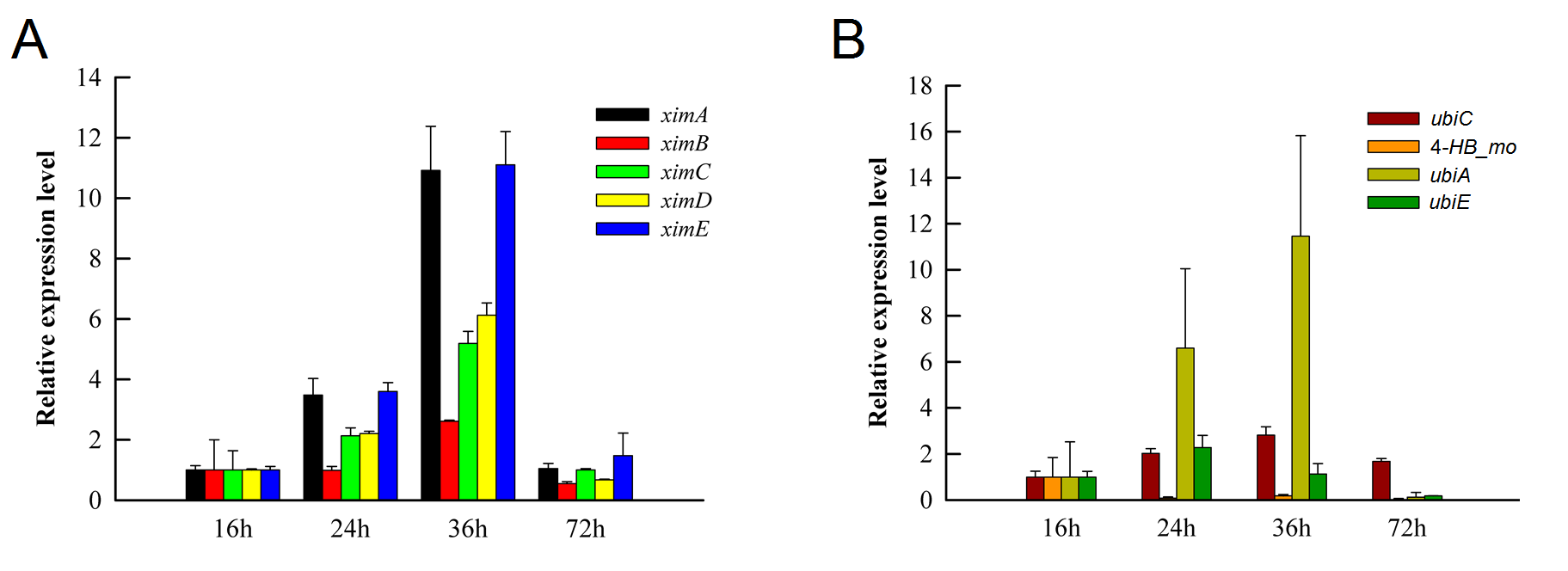


**Supplementary Fig. S5 | Transcriptional levels of the *xim* genes and the *ubi* genes that encode chorismate lyase and prenyltransferase in *S. xiamenensis* 318 at different growth phases.** The relative transcription levels of the target genes were normalized internally to the housekeeping gene *hrdB* (SXIM_45690), which was used as the internal control. A: *ximA* (SXIM_01890), *ximB* (SXIM_01880), *ximC* (SXIM_01870), *ximD* (SXIM_01860), *ximE* (SXIM_01850); B: *ubiC* (SXIM_13770), 4-*HB_mo* (4-hydroxybenzoate 3-monooxygenase, SXIM_13780), *ubiA* (SXIM_13790), *ubiE* (SXIM_13800).


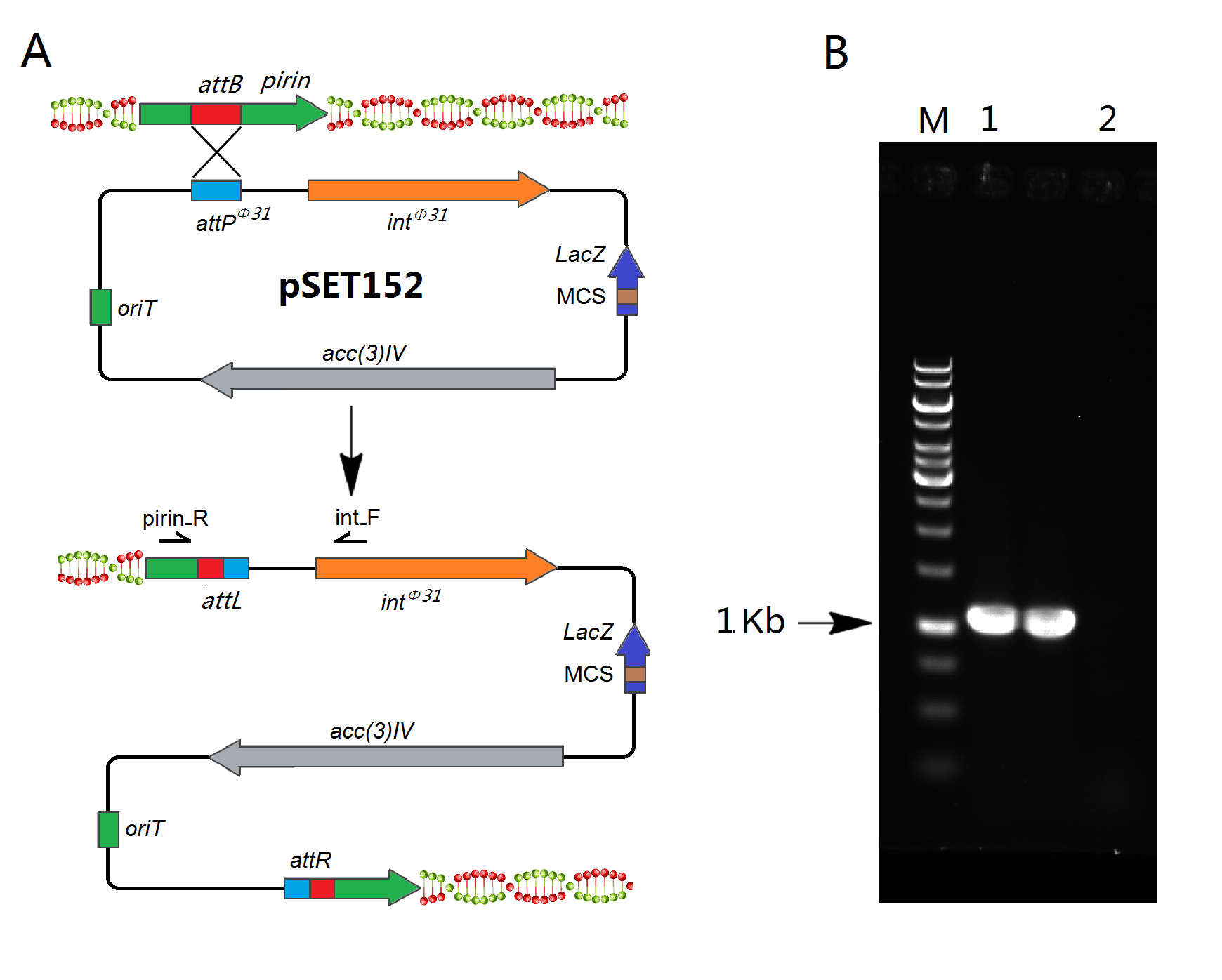


**Supplementary Fig. S6 | Confirmation of *attB* in the chromosome of *S. xiamenensis* 318 by pSET152 integration.** A: Schematic diagram of pSET152 integration. B: M. Thermo GeneRuler 1 kb DNA Ladder; 1. Apramycin-resistant exconjugant *S. xiamenensis* 318::pSET152; 2. *S. xiamenensis* 318 wild-type.


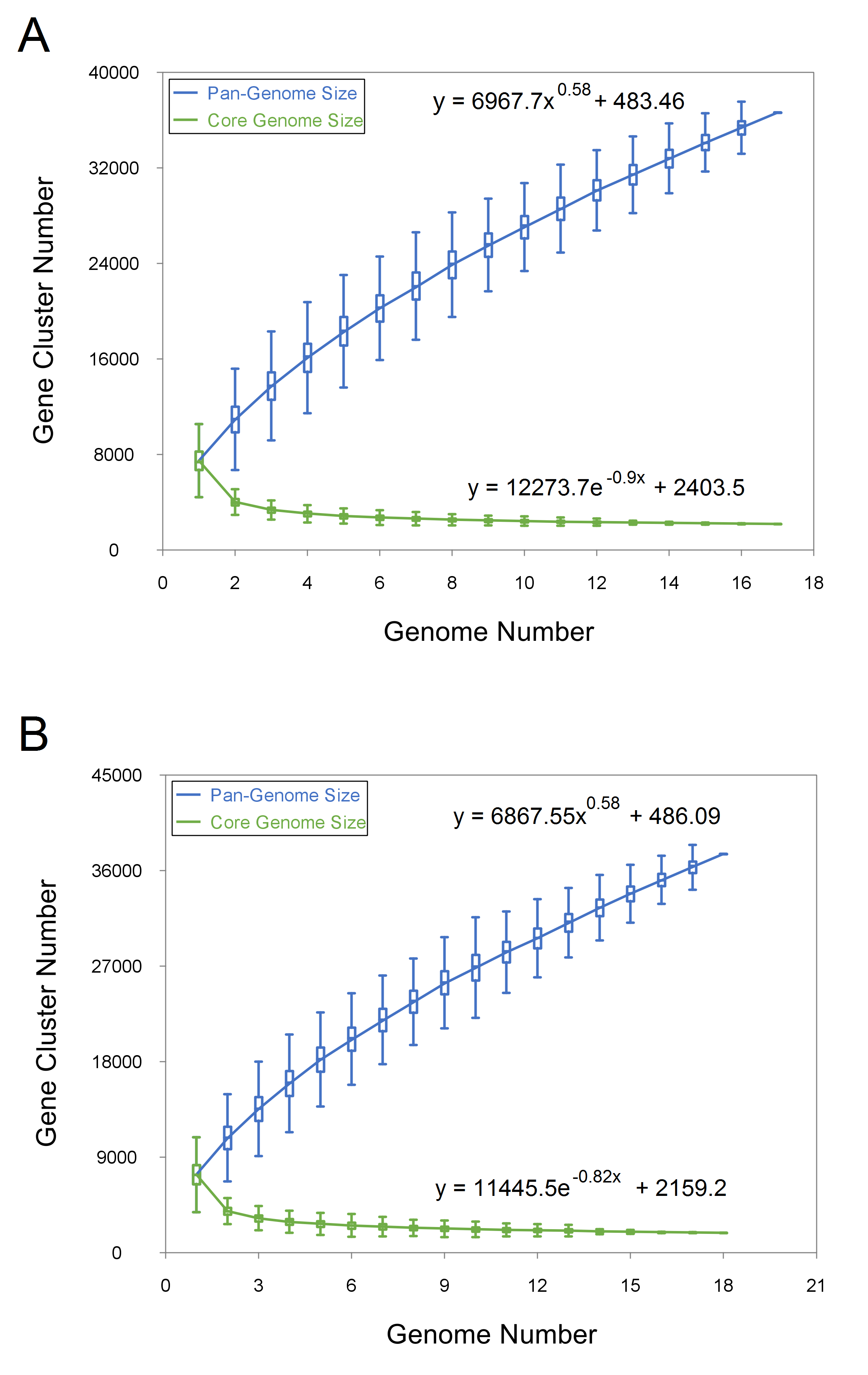


**Supplementary Fig. S7 | Pan-genome analysis of 18 *Streptomyces* species.** The numbers of gene cluster in the *Streptomyces* pan-genome and core genome are plotted against the number of genomes added. The deduced mathematical function is also reported. *A: genomes of 17 *Streptomyces* species. B: genomes of 17 *Streptomyces* species plus *S. xiamenensis* 318. *GenBank Access Nos. of the 17 genomes were reported by Kim J. et al. (2015).
